# Cucurbitacin B alleviates DSS-induced ulcerative colitis by improving gut microbiota disorder in C57BL/6 mice

**DOI:** 10.1186/s13568-025-01922-5

**Published:** 2025-07-26

**Authors:** Jichen Li, Xiaodi Shen, Xia Wu, Fan Zhao, Wenling Tang, Minglan Wu, Fang Hu, Xingjiang Hu, Fei Wang, Qiao Zhang

**Affiliations:** 1 Phase I Clinical Research Center, The First Affiliated Hospital of Zhejiang Chinese Medical University (Zhejiang Provincial Hospital of Traditional Chinese Medicine), Hangzhou, 310006 China; 2 Zhejiang Provincial Key Laboratory of Traditional Chinese Medicine for Clinical Evaluation and Translational Research, Zhejiang Provincial Key Laboratory for Drug Evaluation and Clinical Research, Department of Clinical Pharmacy, The First Affiliated Hospital, Zhejiang University School of Medicine, Hangzhou, 310003 People’s Republic of China; 3 Office of National Drug Clinical Trail Institution, The Fifth Affiliated Hospital, Wenzhou Medical University, Lishui, 323000 People’s Republic of China; 4 Zhejiang Provincial Key Laboratory for Drug Evaluation and Clinical Research, The First Affiliated Hospital, Zhejiang University School of Medicine, Hangzhou, 310003 People’s Republic of China

**Keywords:** Cucurbitacin B, Microbiota, Ulcerative colitis, Dextran sulfate sodium

## Abstract

Cucurbitacin B (CuB) is a triterpenoid compound derived from various medicinal plants, demonstrating potential anti-inflammatory, antioxidant, and neuroprotective properties, as well as significant anti-tumor effects. However, its efficacy in treating ulcerative colitis (UC) remains unclear. To investigate the therapeutic potential of CuB, a dextran sulfate sodium (DSS)-induced UC model in mice was employed, along with gut microbiota analysis. The results revealed that CuB significantly alleviated clinical symptoms, improved colonic tissue damage, and suppressed pro-inflammatory cytokines such as TNF-α, IL-1β, and IL-6 in colon tissues. Additionally, CuB was associated with changes in specific microbial populations, including the upregulation of *Muribaculaceae*, *Rikenellaceae_RC9_gut_group*, *Muribaculum*, and *Bifidobacterium*, and the downregulation of *Desulfovibrionaceae*, *Helicobacter*, *Escherichia-Shigella*, *Streptococcus*, *Candidatus_Saccharimonas*, and *Clostridium*, which may contribute to the recovery of colon injury. This study provides preliminary evidence supporting CuB's therapeutic potential in DSS-induced colitis by enhancing gut microbiota diversity. CuB shows promise as potential treatment for UC and other conditions related to disruptions in intestinal flora homeostasis.

## Key points

- We firstly confirmed the anti-ulcerative colitis effects of CuB in vivo.
- CuB could significantly improve the disorders of intestinal flora.
- CuB’s anti-inflammatory activity is highly correlated with gut microbiota.

## Introduction

Inflammatory bowel diseases (IBD) are chronic immune-mediated diseases of the gastrointestinal tract, typically categorized as Crohn’s disease or ulcerative colitis (UC) based on symptoms and histological characteristics. Ulcerative colitis has emerged as a global health issue, with rapidly increasing incidence in newly industrialized countries that have adopted more westernized lifestyle (Ng et al. 2017). Inflammation, gut microbiota, and condition of immune system were strongly associated with the prevalence and improvement of UC (Wendelsdorf et al. 2010). The connection between the gut microbiota and immune system of mucosa has been considered crucial in chronic inflammation, and the alterations of variety and composition of intestinal microbiome may play a significant role in the treatment of UC. Currently, the clinical treatment of IBD includes non-biological agents, including corticosteroids, immunomodulators, and aminosalicylates, and biological agents like monoclonal antibodies. Each medication has limitations such as limited efficacy, high relapse rates, or high costs, thus there is an urgent need to develop safe, effective, and affordable oral natural medicines.

Precedent research demonstrated that triterpenoids are a class of compounds that could modulate intestinal inflammation in UC by the mechanisms of tuning immune response and gut microbiota (Liu et al. 2015). Cucurbitacin B (CuB), widely distributed in plants such as cucumber, watermelon, and pumpkin, is a naturally occurring tetracyclic triterpenoid compound found in plants of the *Cucurbitaceae* family and is known for its bitterness and diverse biological activities (Ma et al. 2022). It exhibits a variety of pharmacological effects, particularly showing significant potential in anti-oxidant, anti-inflammatory, and antiviral applications (Nie et al. 2024). Previous studies have demonstrated that CuB can modulate inflammatory responses by reducing the release of inflammatory factors, indicating its potential as promising treatment for various inflammation-related diseases (Zhong et al. 2020). Meanwhile, CuB has garnered significant attention for its potential health benefits mainly via regulating various signaling pathways. Several studies have shown that CuB inhibits the JAK2/STAT3 pathway, which is critical for inflammatory response, and affects the NF-κB signaling cascade, which may contribute to its anti-inflammatory properties (Zhang et al. 2017; Kusagawa et al. 2022). However, there are few reports on its research in regulating gut microbiota.

Although the anti-inflammatory activity of cucurbitacins has drawn substantial interest recently, few studies have been conducted on CuB of its anti-UC activity (Dai et al. 2023). In addition, many studies of its toxicity and pharmacokinetic properties showed that CuB has non-specific toxicity and low bioavailability, which highlights the need to investigate the therapeutic effect of CuB on UC. In the meantime, the mechanisms of action remain veiled though cucurbitacins now recognized as the compounds that could regulate immune homeostasis and gut microbiota in vivo. Thus, elucidating the underlying mechanisms is of paramount importance. Our research groups have conducted a large number of studies on the screening of active ingredients from TCM for the treatment of UC (Zhang et al. 2020; Hu et al. 2020; Wu et al. 2022). Based on these results, we attempt to explain the anti-inflammatory mechanism of CuB on UC by starting from a novel mechanism for regulating gut microbiota dysbiosis. This research preliminarily confirmed the effects of CuB on dextran sulfate sodium (DSS) induced UC mice from another perspective of regulating effects of gut micobiota utilizing modern pharmacological techniques. We hope that the mechanism of action of CuB can provide more insights for the application of CuB on other diseases related to gut microbiota disorders.

## Material and methods

### Drugs and reagents

DSS was bought from MP Biomedicals (Irvine, California, USA), as described in our earlier study (Zhang et al. 2020). Salicylazosulfapyridine (SASP) was obtained from Solarbio (Beijing, China). CuB was obtained from Med Chem Express (MCE, Shanghai, China). Enzyme-linked immunosorbent assay (ELISA) kits for mice of TNF-α, IL-1β, and IL-6 were procured from ELK Biotechnology (Wuhan, Hubei, China).

### The design of experiments

Thirty male C57BL/6 SPF mice were acquired from the Experimental Animal Center of Zhejiang Province (Hangzhou, China). The mice were housed under standard conditions with a controlled temperature of 18–23 °C and a twelve-hour light/dark cycle. After one week of acclimatization, the mice were randomly assigned to five groups, with six mice per group. Mice in the control group were provided with ad libitum to pure water, while the remaining groups were provided with a 3% DSS solution for 7 days to induce UC. The DSS solution was replaced with a freshly prepared solution every two days. CuB were administered orally twice daily for 7 days at a volume of 0.1 mL/10 g body weight. Based on prior studies and preliminary experiments (Dai et al. 2023; Nie et al. 2024), the doses of CuB were chosen as 1 mg/kg and 2 mg/kg, while SASP was administered at 200 mg/kg. The control and DSS-only groups received an equivalent volume of saline. The body weight of mice was monitored every day. On the 8th day, all mice were sacrificed by cervical dislocation, and colon tissue samples as well as fecal samples were collected for analysis.

### The evaluation of anti-UC effects

The Disease Activity Index (DAI) score is a well-established metric for evaluating colon injury in experimental animal models. It is commonly used to assess clinical symptoms in mice following DSS administration, including weight loss, diarrhea, fecal bleeding, and other signs of distress. In accordance with the DAI scoring criteria (Hu et al. 2020), daily blinded assessments were conducted to monitor body weight, activity levels, stool consistency, and the presence of blood in feces. On day 8 of the experiment, colons were dissected and measured using a standardized ruler. Subsequently, each colon was divided into two segments: one was fixed for histopathological staining, while the other was immediately snap-frozen in liquid nitrogen for subsequent analysis of inflammatory mediators.

### The evaluation of pathological features of colon tissues

Colon tissues were quickly dissected, measured, and photographed. A portion of the colon tissue was then fixed in 4% paraformaldehyde for 48 h. Then, the colon fragments were embedded, sectioned, stained and observed under the microscope. The histological score was assessed based on infiltration neutrophils and lymphohistiocytes, crypt abscesses, extent of crypt damage, sub-mucosal edema, loss of goblet cells and reactive epithelial hyperplasia, following the scoring criteria outlined in our previous paper (Wu et al. 2022). Colon histopathological improvement was evaluated through calculating sum of each score. As shown in Table 1, the histological score was evaluated following the scoring criteria (Sann et al. 2013).

**Table 1:** Histological scoring system

Score	Extent of Inflammation	Infiltration neutrophils + lympho-histiocytes	Extent of crypt damage	Crypt abscesses	Sub-mucosal oedema	Loss of goblet cells	Reactive epithelial hyperplasia
0	None	None	None	None	None	None	None
1	Mucosa	Focal	Basal one third	Focal	Focal	Focal	Focal
2	Mucosa + submucosa	Multifocal	Basal two third	Multifocal	Multifocal	Multifocal	Multifocal
3	Mucosa + submucosa + muscle layer	Diffuse	Entire crypt damage	NA	Diffuse	Diffuse	Diffuse
4	Transmural	NA	Crypt damage + ulceration	NA	NA	NA	NA

*NA* not applicable

### Biochemical evaluation of the colon tissue

Briefly, colon tissue samples were homogenized in cold phosphate-buffered saline (PBS) or an appropriate lysis buffer, followed by centrifugation at 10,000 × *g* for 10 min at 4 °C to collect the supernatant. The protein concentration of each sample was determined using a BCA protein assay kit to ensure equal loading. The levels of inflammatory factors (TNF-α, IL-1β, and IL-6) in the colon of UC mice were measured using ELISA kits following the instructions (ELK Biotechnology, Wuhan, Hubei, China).

## Gut microbiota analysis

### DNA extractions

Gut microbiota analysis was performed as described previously (Shi et al. 2024). DNA was extracted from fecal samples using the E.Z.N.A.® Stool DNA Kit (D4015, Omega, Inc., Norcross, Georgia, USA). A blank control with nuclear-free water was included during the extraction process. The extracted DNA was eluted in 50 µL of elution buffer for downstream analysis, including PCR and sequencing, which were conducted by LC-Bio Technology Co., Ltd. (Hangzhou, Zhejiang, China).

### 16S rDNA gene sequencing and data analysis

The V4 region of the 16S rRNA gene was amplified using the primers 515F (5′-GTGYCAGCMGCCGCGGTAA-3′) and 806R (5′-GGACTACHVGGGTWTCTAAT-3′). Each PCR reaction was carried out in a 25 µL mixture containing 25 ng of template DNA, 12.5 µL of PCR premix, and 2.5 µL of each primer. The thermocycling conditions included an initial denaturation at 98 °C for 30 s, followed by 35 cycles of denaturation at 98 °C for 10 s, annealing at 54 °C or 52 °C for 30 s, and extension at 72 °C for 45 s. A final extension step was performed at 72 °C for 10 min. The PCR products were purified using AMPure XT beads (Beckman Coulter Genomics, Danvers, MA, USA) and quantified using a Qubit fluorometer (Invitrogen, USA). The prepared amplicon libraries were analyzed for size and quantity using an Agilent 2100 Bioanalyzer (Agilent, USA) and the Library Quantification Kit for Illumina (Kapa Biosciences, Woburn, MA, USA). Sequencing was conducted on the Illumina HiSeq PE150 platform. For data analysis, as the protocol previously reported (Tao et al. 2019), samples were sequenced on the Illumina HiSeq platform according to the manufacturer's instructions (LC-Bio). Paired-end reads were processed by assigning, truncating and merging using FLASH. Chimeric sequences were filtered by using Vsearch software (v2.3.4). Sequences with ≥ 97% similarity were clustered to the identical operational taxonomic units (OTUs) by Vsearch (v2.3.4). Representative sequences for each OTU were selected, and taxonomic classification was performed using the Ribosomal Database Project classifier. To compare differences of the dominant species across groups, multiple sequence alignments were conducted using the mafft software (v7.310) to investigate the phylogenetic relationship of different OTUs. All these indices in our samples were calculated with QIIME (Version 1.8.0). The Illumina sequencing raw data have been deposited in NCBI Sequence Read Archive database (Submission ID: SUB15166341; Bioproject ID: PRJNA1234501).

### Statistical analysis

For the analysis of the intestinal microbiota, splicing sequences was conducted by FLASH 1.2.8 software, while Vsearch 2.3.4 software was utilized for chimeras filtering and OTU clustering, diversity of microflora was assessed using QIIME 1.8.0 software, and R 3.4.4 (R Core team) language mapping software was employed. Other results were represented as mean ± SD of three parallel experiments. The statistical significance between experimental groups was determined by one-way ANOVA followed by Student’s *t*-test or post hoc Tukey’s test when applicable by employing GraphPad 7.0 software.

## Results

### CuB ameliorated the symptoms of UC mice

**Fig. 1:**
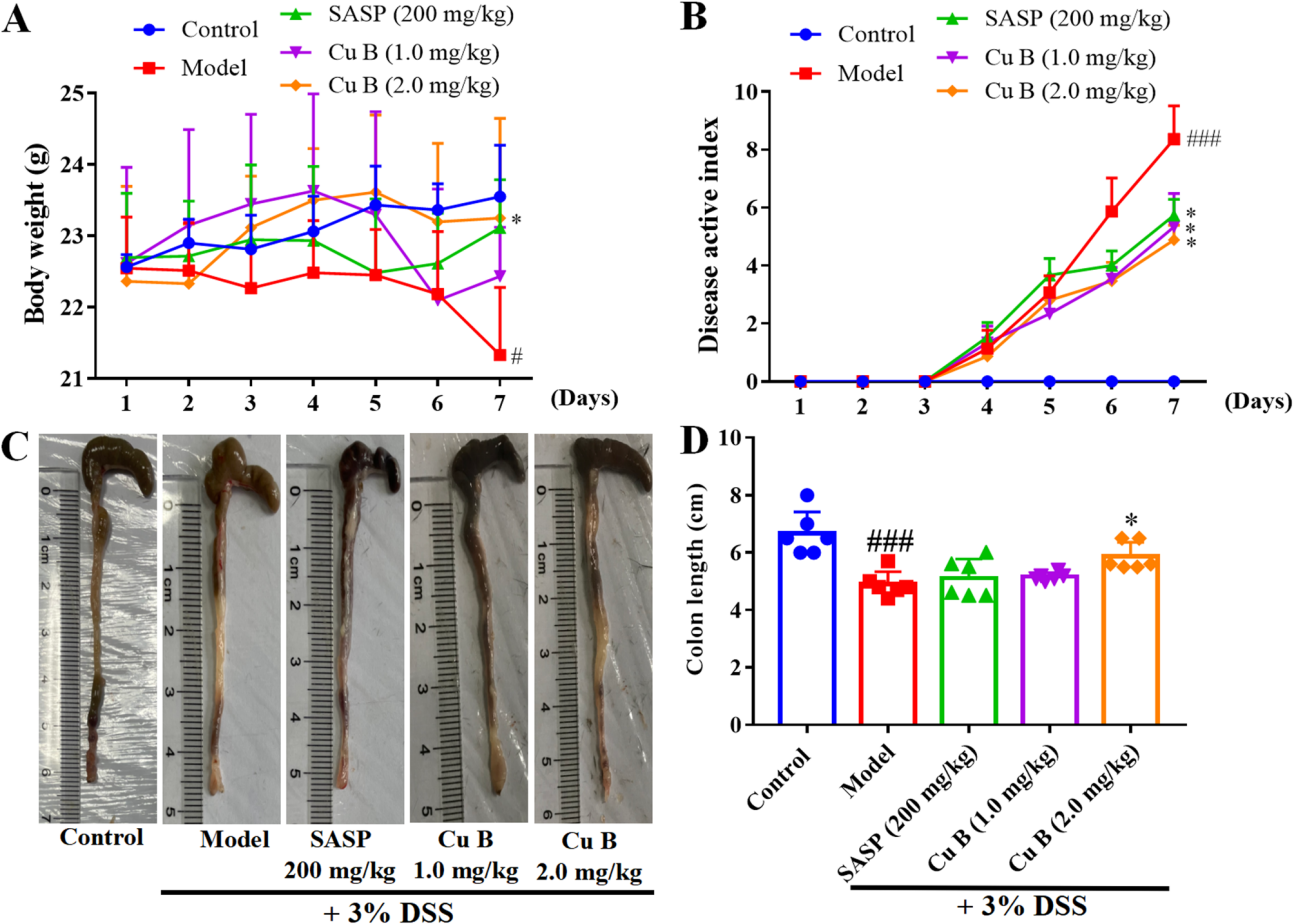
The effect of CuB in colitis tissues. **A** Changes of body weight in mice of different groups. **B** DAI changes in different experimental groups. **C** Improved length of the colon by administration of CuB. **D** Quantitative results of colon length. Each dot represented the mean ± SD (*n* = 6). ^#^*p* < 0.05, ^###^*p* < 0.001 vs. Control; *p < 0.05 vs. Model

DSS significantly reduced the body weight of mice starting on day 5. During the treatment period (days 1–7), mice administered CuB exhibited less weight loss compared to the DSS group (Fig. 1A). Additionally, the DAI scores improved markedly in mice treated with CuB and SASP (Fig. 1B). Colon length, a key indicator of colitis severity, decreased notably in the model group, from 6.67 ± 0.75 cm to 4.90 ± 0.44 cm (Fig. 1C). However, CuB (2.0 mg/kg) significantly restored colon length to 5.87 ± 0.49 cm, counteracting the DSS-induced shortening (Fig. 1D).

### CuB mitigated the colonic damage of DSS-induced colitis mice

**Fig. 2:**
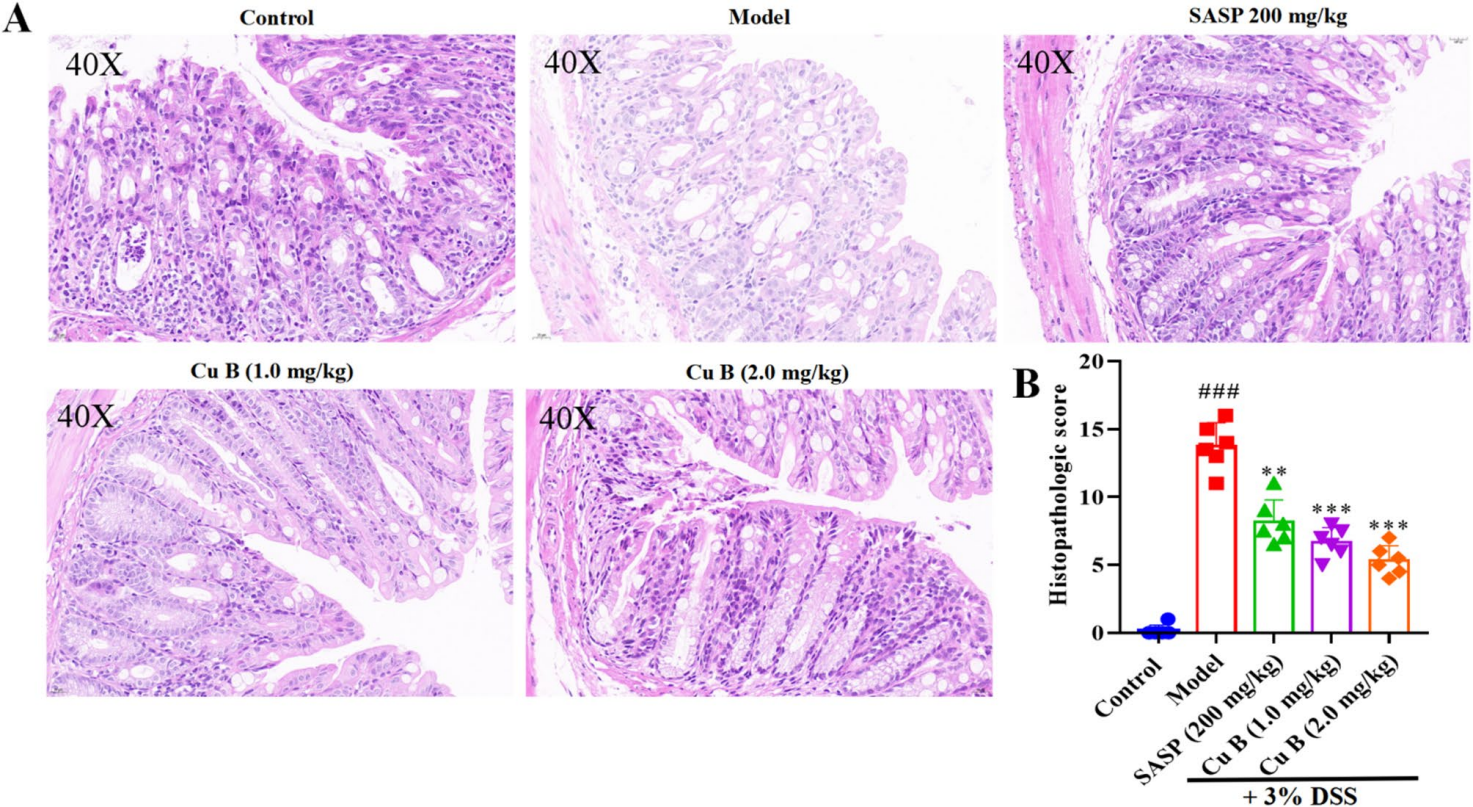
H&E-stained of representative cross-sectional colon tissues in DSS-induced mice after the treatment of CuB for seven days. **A** Representative photos of colonic sections. **B** Morphology score. Each dot represented the mean ± SD (*n* = 6). ^###^*p* < 0.001 vs. Control; **p < 0.01 and ***p < 0.001 vs. Model

Control group of mice exhibited intact colon structure, with a clear and complete distribution of the mucosa, submucosa, muscularis, and outer membrane (Fig. 2A). In the model group, DSS treatment caused significant colonic damage, including epithelial monolayer erosion, crypt loss, and immune cell infiltration, observed in the submucosa and muscular layer of the colon, which indicated by significantly increased histopathologic score comparing to the control group (Fig. 2B). Compared to the DSS group, the histopathological scores in SASP and CuB groups were remarkably reduced, with CuB eliciting better efficacy than SASP, particularly the high doses. In SASP (200 mg/kg) and CuB (1 and 2 mg/kg) group, inflammatory infiltration was mildly ameliorated, and only a few gland pits and goblet cells were visible.

### CuB alleviated DSS-induced the elevation of inflammatory factors in colon

**Fig. 3:**
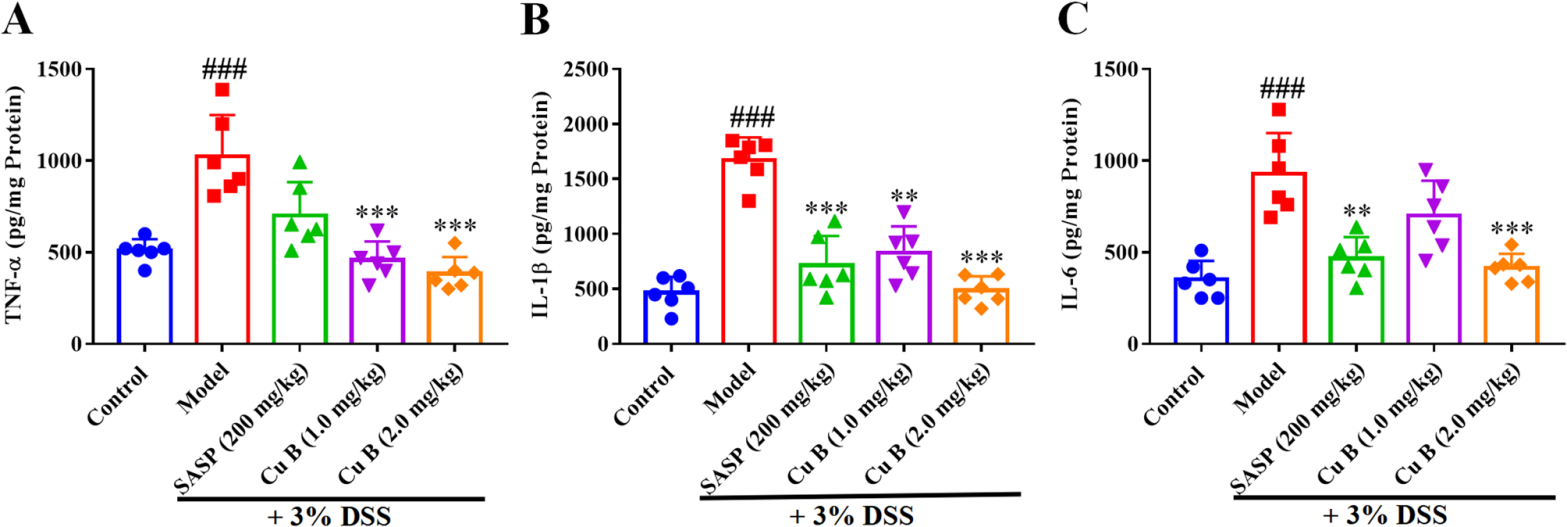
The inhibition of pro-inflammatory cytokine levels in colon tissues by CuB. **A** TNF-α levels; **B** IL-1β levels; **C** IL-6 levels. Each dot represented the mean ± SD (*n* = 6). ^###^*p* < 0.001 vs. Control; **p < 0.01 and ***p < 0.001 vs. Model

Comparing to mice of the control group, DSS treatment resulted in the up-regulation of various pro-inflammatory cytokines of TNF–α (Fig. 3A), IL–1β (Fig. 3B) and IL–6 (Fig. 3C) in colon tissues. The concentrations of TNF–α, IL–1β and IL–6 increased from 508.37 ± 64.01 to 1091.00 ± 368.19, 468.33 ± 144.14 to 1672.33 ± 204.87 and 352.73 ± 100.71 to 928.20 ± 222.81 pg/mg protein of the DSS group, respectively. Consistent with the anti–UC effect above, the decreases of these three inflammatory factors were 384.33 ± 89.75, 488.40 ± 126.81 and 415.07 ± 77.37 pg/mg protein after CuB (2.0 mg/kg) treatment.

## Microbial diversity analysis

### ɑ-Diversity analysis

**Fig. 4:**
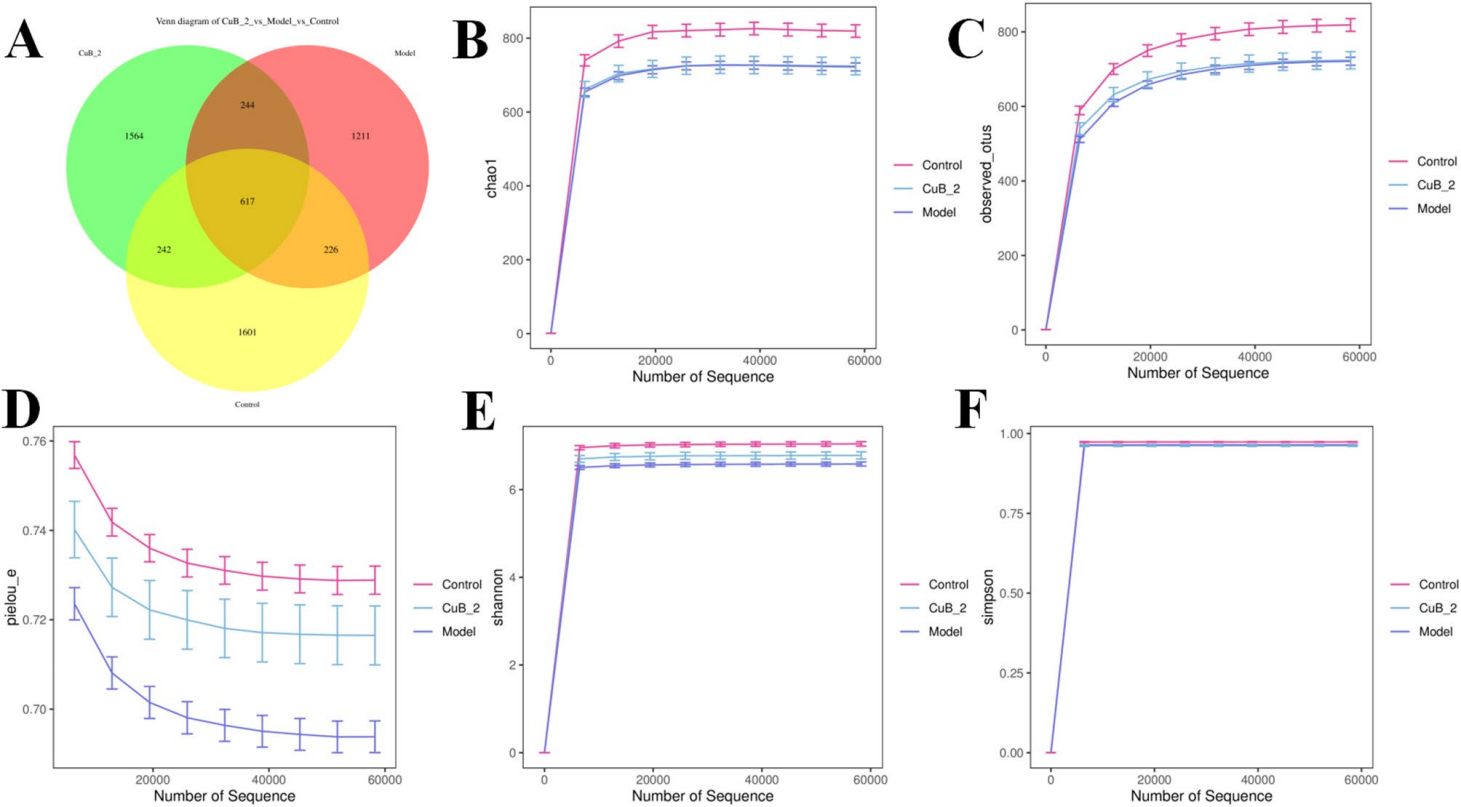
Analysis ɑ-diversity of the differential microbial community among different groups. **A** Venn diagram; **B** Chao1; **C** observed species index; **D** pielou_e; **E** Shannon; **F** Simpson

As illustrated in the Venn diagram of Fig. 4A, 5705 OTUs were common to all groups, while 843 OTUs overlapped in control and model groups, suggesting that significant decrease of diversity of intestinal bacteria caused by DSS. 859 OTUs overlapped in the control and CuB groups, suggesting that CuB could partially restore bacterial diversity. The OTUs of individual control, model, and CuB_2 group were 1601, 1211 and 1564, respectively. These findings suggested that DSS alone significantly diminished intestinal microbiota diversity, whereas CuB_2 treatment improved it to some extent. The rarefaction curves reached a saturated plateau, indicating sufficient sequencing coverage for further analysis. As shown in Table 2, the results from the microbial richness (Chao1) (Fig. 4B) and observed species index (Fig. 4C) revealed a significant reduction in alpha diversity between the control and model groups, which was largely reversed by CuB_2 treatment. The Pielou_e (Fig. 4D), Shannon (Fig. 4E) and Simpson (Fig. 4F) indices also showed a similar trend of changes. These results showed that CuB_2 could significantly enhance microbial diversity in DSS-treated mice.

**Table 2:** The results of alpha_diversity analysis

	Control	Model	CuB_2
Chao1	819.49 ± 144.11	722.63 ± 90.09	723.95 ± 197.18
Observed_otus	818.67 ± 144.24	721.50 ± 90.05	723.50 ± 196.91
Shannon	7.04 ± 0.43	6.58 ± 0.39	6.78 ± 0.68
Simpson	0.97 ± 0.01	0.96 ± 0.01	0.96 ± 0.02
Pielou_e	0.73 ± 0.03	0.69 ± 0.03	0.72 ± 0.06

### β-diversity and cluster analysis

**Fig. 5:**
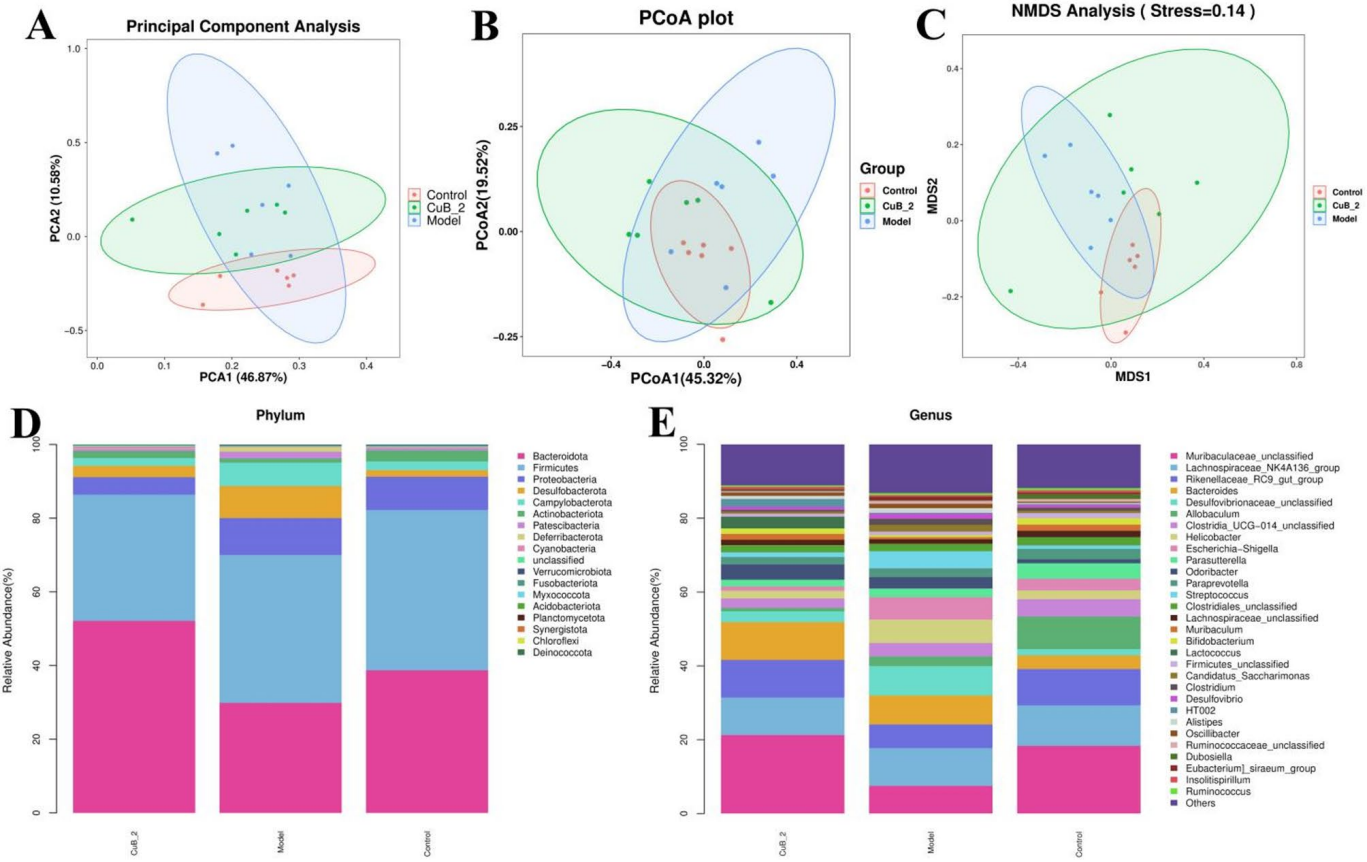
Analysis of the bacterial community diversity in intestinal contents among control, model and CuB_2 groups. **A** Weighted PCA analysis based on UniFrac distance. **B** Unweighted PCoA analysis according to UniFrac distance. **C** Weighted NMDS analysis based on UniFrac distance. Bacterial taxonomic profiling at the phylum level (**D**) and genus level (**E**)

Principal component analysis (PCA) and principal coordinate analysis (PCoA) revealed partial overlap between the control and model groups, indicating a high number of differential OTUs (Fig. 5A, B). However, CuB_2 groups were more closely aligned with the control, suggesting that CuB_2 group’s capability to restore the microbiota towards a normal state. The results of non-measurement multidimensional scaling analysis (NMDS) also illustrated distinct clustering between the model and control groups, reflecting variations in the major microbial composition (Fig. 5C). However, the community structure of CuB_2 groups was significantly inclined to the control group. Metastatistical analysis showed that, at the phylum level, *Desulfobacterota, Campylobacterota,* and *Patescibacteria* exhibited significant increase in the relative abundance of model group (Table 3). However, CuB_2 exhibited significant decreases of these changes of these four phyla (Fig. 5D). At the genus level, CuB_2 reversed the decrease of *Muribaculaceae* and *Rikenellaceae_RC9_gut_group,* as well as the increase of *Desulfovibrionaceae, Helicobacter, Escherichia-Shigella,* and *Streptococcus* in the model (Fig. 5E). However, CuB_2 notably counteracted the levels of these changes (Table 4).

**Table 3:** Cluster analysis of phylum level

Phylum	Control	Model	CuB_2
*p_Bacteroidota*	38.72 ± 8.86	29.87 ± 11.46	52.15 ± 23.23
*p_Firmicutes*	43.52 ± 10.93	40.15 ± 9.06	34.22 ± 20.52
*p_Proteobacteria*	9.04 ± 3.40	10.04 ± 7.81	4.76 ± 1.91
*p_Desulfobacterota*	1.76 ± 1.08	8.67^#^ ± 7.46	3.09* ± 1.78
*p_Campylobacterota*	2.37 ± 0.97	6.33^#^ ± 4.97	2.11* ± 1.76
*p_Actinobacteriota*	2.97 ± 0.86	1.18 ± 0.52	1.90 ± 3.40
*p_Patescibacteria*	0.63 ± 0.53	1.82^#^ ± 1.47	0.60* ± 0.80
*p_Deferribacterota*	0.10 ± 0.10	1.22^#^ ± 1.44	0.14* ± 0.14
*p_Cyanobacteria*	0.23 ± 0.16	0.18 ± 0.10	0.50 ± 0.37
*p_unclassified*	0.31 ± 0.11	0.22 ± 0.11	0.33 ± 0.22

Data were presented as mean ± SEM, *n* = 6, ^#^*p* < 0.05 vs. Control; *p < 0.05 vs. Model

**Table 4:** Cluster analysis of genus level

Genus	Control	Model	CuB_2
*g_Muribaculaceae_unclassified*	18.31 ± 6.94	7.50^#^ ± 3.01	21.27* ± 11.83
*g_Rikenellaceae_RC9_gut_group*	9.82 ± 4.84	6.42 ± 4.20	10.18 ± 6.13
*g_Desulfovibrionaceae_unclassified*	1.69 ± 1.05	7.95^#^ ± 6.42	2.91* ± 1.62
*g_Helicobacter*	2.37 ± 0.97	6.34^#^ ± 4.98	2.11* ± 1.77
*g_Escherichia-Shigella*	3.12 ± 2.99	6.07^#^ ± 6.64	1.14* ± 1.11
*g_Streptococcus*	0.89 ± 0.21	4.63^#^ ± 4.02	1.26* ± 0.78
*g_Muribaculum*	1.66 ± 0.74	0.57^#^ ± 0.38	1.58* ± 0.70
*g_Bifidobacterium*	1.77 ± 0.45	0.56^#^ ± 0.29	1.46* ± 2.92
*g_Candidatus_Saccharimonas*	0.62 ± 0.53	1.81^#^ ± 1.47	0.60* ± 0.80
*g_Clostridium*	0.85 ± 0.46	1.66^#^ ± 1.50	0.47* ± 0.34
*g_Desulfovibrio*	0.72 ± 0.88	1.37 ± 1.97	0.82 ± 0.81
*g_Alistipes*	0.30 ± 0.17	1.26^#^ ± 1.07	0.88 ± 0.72
*g_Oscillibacter*	0.35 ± 0.26	1.15^#^ ± 1.26	0.88 ± ± 0.98
*g_Eubacterium]_siraeum_group*	0.44 ± 0.58	0.95 ± 0.66	0.27* ± 0.34
*g_Others*	11.83 ± 1.50	13.18 ± 2.63	11.12 ± 2.52

Data were presented as mean ± SEM, *n* = 6, ^#^*p* < 0.05 vs. Control; *p < 0.05 vs. Model

### CuB treatment altered overall structure of gut microbiota

**Fig. 6:**
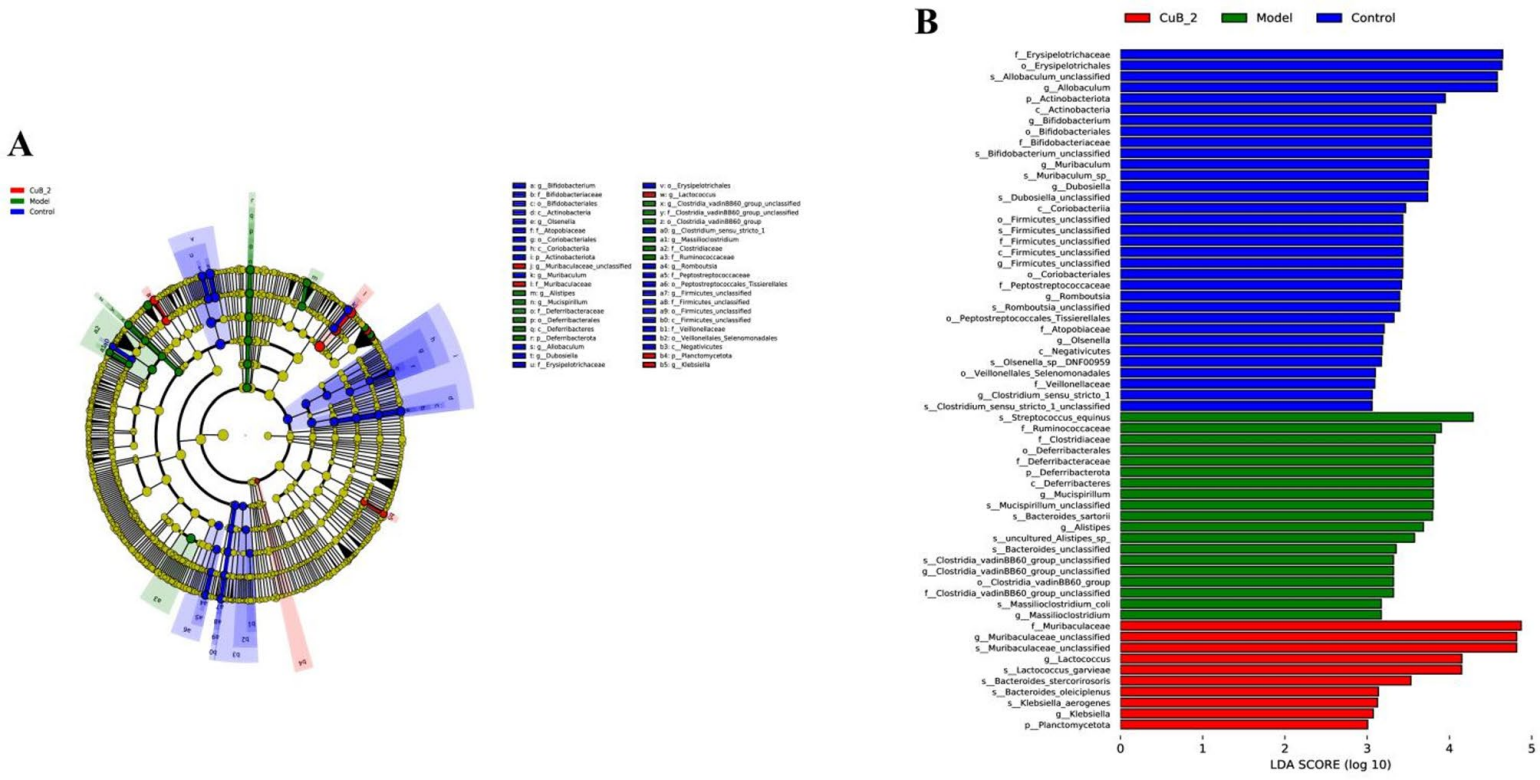
Comparison of the structural composition of fecal flora across the control, model, and CuB_2 groups. **A** Cladogram. The size of every node represents the relative abundance of the species. (p, phylum; c, class; o, order; f, family; g, genus; s, species). **B** LEfSe analysis displayed a distribution histogram of the gut microbiota across different groups (LDA sore > 3.0)

The linear discriminant analysis effect size (LEfSe)-generated cladogram (LDA score > 3.0) highlighted distinct microbial taxa enriched in each group. The radial tree depicted phylogenetic hierarchies (phylum to genus), with colored circles indicating discriminative taxa (circle size = relative abundance). Specifically, the control group was dominated by *f_Bifidobacteriaceae* and *g_Bifidobacterium*, the model group by *p_Deferribacterota* and *f_Deferribacteraceae*, and the CuB_2 group by *f_Muribaculaceae* and *g_Muribaculaceae_unclassified* (Fig. 6A). Consistent with the above results, DSS reduced the relative abundance of *d__Bacteria* *p__Bacteroidota*|* c__Bacteroidia*|* o__Bacteroidales*|* f__Muribaculaceae*|* g__Muribaculaceae_unclassified*, *d__Bacteria*|* p__Bacteroidota*|* c__Bacteroidia*|* o__Bacteroidales*|* f__Rikenellaceae*|* g__Rikenellaceae_RC9_gut_group* while comparing dominant bacterial taxa at genus level. Meanwhile, DSS increase the abundances of *d__Bacteria*|* p__Campylobacterota*|* c__Campylobacteria*|* o__Campylobacterales*|* f__Helicobacteraceae*|* g__Helicobacter*, *d__Bacteria*|* p__Proteobacteria*|* c__Deltaproteobacteria*|* o__Desulfovibrionales*|* f__Desulfovibrionaceae*|* g__Desulfovibrio*, *d__Bacteria*|* p__Proteobacteria*|* c__Gammaproteobacteria*|* o__Enterobacterales*|* f__Enterobacteriaceae*|* g__Escherichia-Shigella*, and *d__Bacteria*|* p__Firmicutes*|* c__Bacilli*|* o__Lactobacillales*|* f__Streptococcaceae*|* g__Streptococcus*. However, CuB_2 significantly reversed the abundances of these main bacterial genus (Fig. 6B). These results further supported our findings in cluster analysis that CuB_2 regulates microbial diversity at both phylum and genus levels in UC mice.

### The correlation analysis of microbiota and inflammatory factors

**Fig. 7:**
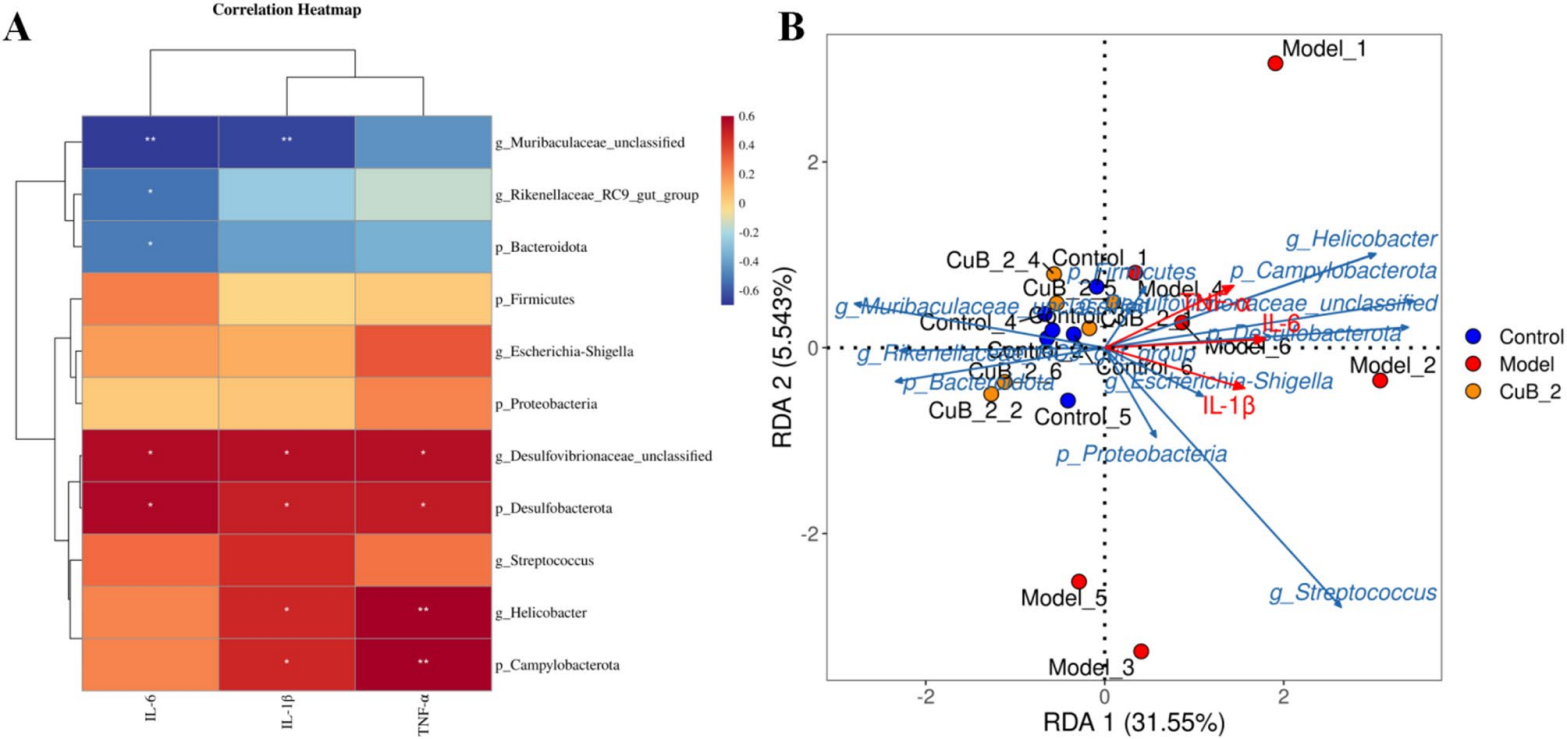
Correlation between gut microbiota and inflammatory factors of different groups. **A** Correlation heatmap analysis; **B** RDA analysis

Most of the bacterial genera upregulated by CuB_2 are negatively correlated with these inflammatory indicators, such as beneficial bacteria of *Muribaculaceae_unclassified* and *Rikenellaceae_RC9_gut_group.* In addition, the conditional pathogenic bacteria and harmful bacteria of *Helicobacter, Desulfovibrionaceae_unclassified, Escherichia-Shigella* and *Streptococcus* were significantly positively correlated with TNF-ɑ, IL-1β and IL-6 (Fig. 7A). Redundancy analysis (RDA) was further conducted to evaluate the correlation between colon cytokine levels and gut microbiota in each group (Fig. 7B). Consistent with the previous findings, the model group formed a distinct cluster separated from the control and CuB_2 groups, suggesting that inflammatory cytokines significantly influenced microbial changes in the disease state, as reflected by their alignment along the same vector directions. CuB_2 also showed a negative correlation with these inflammatory markers, as well as with conditionally pathogenic and harmful bacteria. Conversely, CuB_2 exhibited a positive correlation with beneficial bacteria such as *Muribaculaceae_unclassified* and *Rikenellaceae_RC9_gut_group*. These statistical results demonstrated a strong association between inflammation-related indicators and gut microbiota composition after CuB_2 treatment.

## Discussion

Triterpenoids are the primary group of anti-inflammatory ingredients that are rich in *Cucurbitaceae* family (Cao et al. 2021) and CuB was a major ingredient of triterpenoid extracted from *Cucurbitaceae* plants, such as *Cucurbita pepo cv Dayangua* (Aribi et al. 2013). The animal models of inflammatory diseases have demonstrated that treatment with the analogues of CuB (Cucurbitacin IIb and E) leads to a significant alteration in gut microbial diversity, promoting the growth of beneficial bacteria and alleviating colitis (Zhan et al. 2024; Zhao et al. 2024). This shift in microbiota composition is associated with a decrease in pro-inflammatory cytokines and markers, thereby alleviating symptoms of inflammation. These results underscore the potential of CuB as a modulator of gut microbiota with significant anti-inflammatory properties and provided strong theoretical support for the anti-UC effects of CuB. Our present research suggested that CuB relieved the symptoms like body weight and length of colon in DSS-induced UC mice and alleviated their colonic injury via modulating of gut microbiome. We originally discovered that CuB could exert therapeutic effects on UC by regulating gut microbiota and anti-inflammatory response, which expands the use of CuB in treating gut microbiota disorders and inflammatory diseases.

Dysbiosis, characterized by an imbalance in the gut microbiota, has been associated with mutiple health conditions, including metabolic disorders and inflammatory bowel diseases (DeGruttola et al. 2016). Gut microbiota plays a crucial role in maintaining human health by participating in metabolic processes, synthesizing vitamins, and protecting against pathogens, especially playing an important role in inflammatory bowel disease and UC (Hou et al. 2022). Therefore, the modulation of gut microbiota presents an auspicious strategy for the prevention and treatment of UC. Research has shown that cucurbitacins can alter the composition and diversity of gut microbiota and CuB's anti-inflammatory properties may further improve gut health by reducing inflammation in the intestinal environment, thus promoting a more favorable microbial composition (Dai et al. 2023). Cytokines like TNF-α are mediators of the intestinal inflammatory processes, involving in the pathogenesis of IBD. As previously discovered, CuB suppresses IL-1β excretion by interfering NLRP3 inflammasome formation and suppresing key glycolytic enzymes in macrophages (Xue et al. 2021). We demonstrated that CuB mitigated the DSS-induced increase in inflammatory factors in colon, including IL-1β, TNF-α and IL-6, which is consistent with previous study. This suggests that the anti-inflammatory effects of CuB and its modulation of gut microbiota may work synergistically. In our current research, there is a significant correlation between the bacterial genera regulated by CuB and the inhibited inflammatory factors. These results suggested that CuB may exert anti-inflammatory and anti-UC effects by regulating gut microbiota, or both mechanisms may coexist and have complementary effects.

The changes of intestinal flora are closely connected with the improvement of UC symptoms, suggesting that CuB may exert therapeutic effects by reshaping the balance of intestinal microbiota. *Muribaculaceae* and *Rikenellaceae RC9_gut_group* are newly discovered microbiota closely related to intestinal health in recent years. They can produce short-chain fatty acids, such as acetic acid, propionic acid, and butyric acid, which have various physiological functions such as anti-inflammatory, maintaining intestinal barrier integrity, and regulating immune response (Xu et al. 2021). *Muribaculum* is a newly discovered butyrate producing bacterium whose increased abundance helps improve intestinal inflammation and repair the intestinal barrier. In addition, *Muribaculaceae* significantly reduced in different colitis mice models, while recovered after anti-UC treatment (Zhu et al. 2024). *Bifidobacterium* is a recognized probiotic that can inhibit the growth of pathogens, regulate immune responses, and promote intestinal barrier function. Upregulation of these beneficial bacterial communities by CuB may exert therapeutic effects on UC by increasing the production of short-chain fatty acids, inhibiting inflammatory responses, and enhancing intestinal barrier function. Our findings demonstrated that CuB could significantly elevate the proportion of *Muribaculaceae, Rikenellaceae_RC9_gut_group, Muribaculum,* and *Bifidobacterium*, suggesting the upregulating of these beneficial bacterial communities is at least one of the mechanisms by which CuB functions.

Meanwhile, CuB downregulates many potential pathogenic bacteria, which may alleviate intestinal inflammation and damage by reducing toxin production, inhibiting bacterial invasion, and regulating immune responses. The presence of *Desulfovibrionaceae* is increased in UC*,* whose positivity was substantially increased in acute and chronic UC at various levels within the colon. *Desulfovibrionaceae* is a sulfate reducing bacterium whose metabolite hydrogen sulfide is toxic to intestinal epithelial cells, disrupting intestinal barrier function and inducing inflammatory responses (Rowan et al. 2010). *Helicobacter* and *Escherichia-Shigella* are common opportunistic pathogens that can produce toxins and invade intestinal epithelial cells, leading to intestinal inflammation and damage. *Escherichia-Shigella*, a leading cause of bacterial diarrhea worldwide, is considered to exacerbate the pro-inflammatory immune responses in IBD. Specific species of *Helicobacter* were also found to be increased in subjects with Crohn's disease and UC compared to members of the control population. (Sasaki and Klapproth 2012). The microbiota of *Streptococcus*, *Candidatus_Saccharimonas*, and *Clostridium* are also associated with intestinal inflammation and metabolic disorders. *Streptococcus* has been strongly associated with gut inflammation, positively correlated with circulating biomarkers related to systemic inflammation and immune response to infection (Sayols-Baixeras et al. 2023). Similarly, evidence from preceding study suggests a strong association between *Clostridium* and gut inflammation (Seicean et al. 2014). These results indicated that reducing the abundance of harmful bacteria is also one of the mechanisms by which CuB functions.

CuB has been acknowledged for its notable anti-inflammatory and immune-modulating effects in animal models, potentially through the NF-κB and Nrf2/HO-1 pathways (Lou et al. 2024). Further studies are needed to elucidate the specific pathways through which CuB exerts its therapeutic effects in UC. Like other cucurbitacins, CuB exhibits non-selective toxicity by interfering with actin filaments (Hohmann and Dehghani 2019). However, when administered at appropriate doses and durations, CuB demonstrates limited toxicity to different cell-lines or animals (Dai et al. 2023). Pharmacokinetic research has proved that CuB can be absorbed and eliminated in vivo and is widely distributed with a high tissue-to-plasma ratio, while with relatively low oral bioavailability (Wang et al. 2017). Given this, oral administration of CuB could potentially maintain higher concentrations at the target site in the intestine, thus enhancing its therapeutic effects for treating UC. As a naturally occurring compound, CuB is readily available and can be used either alone or in combination with other therapies. Therefore, future efforts should focus on modifying its chemical structure of CuB, developing novel drug delivery system, and exploring its combination with other drugs to minimize toxicity and enhance its clinical applications. Combined with our results, CuB has great research potential in the treatment of UC and detailed mechanisms of CuB effect on UC need to be confirmed by further experiments, as well as human clinical trials.

In summary, CuB elicited promising treatment efficacy on DSS-induced UC mice. These therapeutic effects included the amelioration of general symptoms and the alleviation of pathological injury in the colon. The underlying mechanism involved preventing intestinal microbiota imbalance, restoring the relative abundances of key bacterial populations and alleviating inflammation response. In conclusion, this study revealed that CuB protects against DSS-induced UC by regulating the composition of the intestinal microbiota.

## Data Availability

No datasets were generated or analysed during the current study.

## Abbreviations

CuB: Cucurbitacin B
DAI: Disease activity index
DSS: Dextran sulfate sodium
ELISA: Enzyme-linked immunosorbent assay
IBD: Inflammatory bowel disease
LEfSe: Linear discriminant analysis effect size
NMDS: Non-metric multidimensional scaling analysis
OTUs: Operational taxonomic units
PBS: Phosphate buffer saline
PCA: Principal component analysis
PCoA: Principal coordinate analysis
SASP: Salicylazosulfapyridine
TCM: Traditional Chinese medicine
UC: Ulcerative colitis
